# A rare case of low-dose methotrexate toxicity leading to skin and mucosal toxicity and invasive pulmonary mucormycosis: a case report

**DOI:** 10.3389/fphar.2025.1704810

**Published:** 2025-11-19

**Authors:** Lin Luo, Lei Wang, Xiaoxia Zhao, Na Zhang, Yue Zhang, Xinggong Xue, Hui Li

**Affiliations:** 1 Shandong University of Traditional Chinese Medicine, Jinan, Shandong, China; 2 Department of Emergency Intensive Care Medicine Center, The Affiliated Hospital of Shandong University of Traditional Chinese Medicine, Jinan, Shandong, China; 3 The Affiliated Tai’an City Central Hospital of Qingdao University, Tai’an, Shandong, China

**Keywords:** idiosyncratic reaction, low-dose methotrexate, mucocutaneous ulcer, impaired immunity, opportunistic infections

## Abstract

Methotrexate is a potentially toxic antifolate antineoplastic and immunosuppressive agent that is widely used in tumor chemotherapy and autoimmune diseases (such as psoriasis). It is the cornerstone therapy for immune-mediated disorders worldwide. However, low-dose methotrexate therapy for psoriasis rarely causes toxicity. Here, we report a case of a patient with psoriasis who was treated with low-dose methotrexate for the first time. The patient developed severe mucosal ulcers and myelosuppression, leading to impaired immunity and invasive pulmonary mucormycosis. After treatment, the patient recovered and was discharged. It demonstrates that even low-dose methotrexate prescribed for psoriasis can induce severe toxicity. The case highlights the potential for low-dose methotrexate toxicity, suggesting that genetic polymorphisms may increase the risk of toxicity. The influence of genetic polymorphisms on methotrexate metabolism is highly variable. It is important to strengthen early monitoring in clinical practice, improve toxicity prediction models, and establish risk assessment systems for toxic drugs.

## 1 Introduction

Methotrexate is 4-amino-N10-methylpteroylglutamic acid, with a structure highly similar to that of folic acid, and it exerts its effects by competitively binding to specific targets (Urbančič et al., 2025). It serves as a cornerstone agent in the treatment of various autoimmune diseases and hematologic malignancies (Tezgin et al., 2024). Methotrexate functions by competitively inhibiting dihydrofolate reductase (DHFR), which prevents the conversion of dihydrofolate to tetrahydrofolate, thereby disrupting DNA synthesis and profoundly impairing the function of rapidly dividing cells. High-dose methotrexate (HD-MTX; typically defined as >500 mg/m^2^) induces acute toxicity that is primarily mediated by the direct inhibition of cellular proliferation and metabolic processes, with nephrotoxicity being the most prominent manifestation; other adverse effects include hepatotoxicity, myelosuppression, gastrointestinal toxicity, and pulmonary toxicity (Howard et al., 2016). The toxicity profile of low-dose methotrexate (LD-MTX, 5 mg–25 mg/week) is mainly related to long-term cumulative effects and alterations in immune regulation. The most frequently reported mild adverse reactions include painful stomatitis and gastrointestinal discomfort. At these low doses, the overall treatment regimen is considered relatively safe. Serious toxicities such as hepatotoxicity, myelosuppression, pulmonary toxicity, and nephrotoxicity are infrequently observed (Romão et al., 2014).

Here, we describe a case of a 69-year-old man with a 40-year history of psoriasis who received methotrexate for the first time. Following the second weekly low-dose methotrexate administration (cumulative dose: 20 mg), he developed explosive mucocutaneous ulceration, followed by myelosuppression and immunosuppression. These complications predisposed him to opportunistic infections and rare invasive pulmonary mucormycosis. We approximately assessed the likelihood of the clinical presentation being secondary to an adverse drug reaction using the Naranjo Adverse Drug Reaction Probability Scale (Naranjo et al., 1981), yielding a score of 9/13 (Table 1). After 51 days of hospitalization, he was finally stabilized and discharged. The patient had no prior history of methotrexate toxicity. This acute reaction to low-dose methotrexate may be explained by genetic polymorphisms affecting drug clearance. Such genetic variations can impair methotrexate elimination, prolong systemic exposure, and intensify the toxic effects. This case highlights the unique nature of toxic drug responses in populations with idiosyncratic reactions, and precision medicine can effectively reduce the risk of severe adverse events.

**TABLE 1:** Naranjo Assessment Scale.

Clinical question	Yes	No	Do not know	Result
1 Are there any previous solid reports on this reaction?	+1	0	0	+1
2 Did the adverse event occur after the suspected agent was administered?	+2	-1	0	+2
3 Did the adverse reaction resolve once the drug had been stopped or a specific antagonist was administered?	+1	0	0	+1
4 Did the adverse reaction develop after the agent was re-administered?	+2	-1	0	0
5 Are there any additional reasons that could have caused the reaction?	−1	+2	0	+2
6 Did the reaction reappear when a placebo was given?	−1	+1	0	+1
7 Was the agent detected in any body fluid in toxic concentration?	+1	0	0	+1
8 Was the reaction worse when the dose was raised or less severe when the dose was reduced?	+1	0	0	0
9 Did the patient react similarly to the same or similar agents in earlier exposures?	+1	0	0	0
10 Was the adverse event supported by objective evidence?	+1	0	0	+1
				Total score 9

Naranjo Assessment Scale consists of 10 clinically relevant questions related to adverse events (AEs), each with the corresponding scoring criteria. After evaluating each item, the total score is classified into four distinct categories: “definite (≥9 points),” “probable (5–8 points),” “possible (1–4 points),” and “doubtful (≤0 points).” This classification is utilized to determine the likelihood of a causal relationship between the AE and the suspected pharmacological agent.

## 2 Manuscript formatting

### 2.1 Case description

A 69-year-old male went to the local hospital due to fever and oral ulceration. His medical history was significant for type 2 diabetes mellitus, end-stage renal disease on hemodialysis for 2 years, and a 40-year history of psoriasis. Two weeks ago, he had presented to a dermatology hospital for severe psoriasis. He was initiated on low-dose methotrexate therapy at 10 mg/week, with concomitant folic acid supplementation at 10 mg/week. Three days ago, he received the second dose (cumulative methotrexate dose, 20 mg) and developed fever and sore throat. One day ago, he developed oral ulcers, facial swelling, dysphagia, and dysarthria. He was transferred to a nearby hospital, where laboratory tests (Table 2) revealed leukocytes of 0.24 × 10^9^/L (3.5–9.5 × 10^9^/L), red blood cells (RBCs) of 3.2 × 10^12^/L (4.3–5.8 × 10^12^/L), a platelet count of 42 × 10^9^/L (125–350 × 10^9^/L), and a high-sensitive C-reactive protein level of 231.09 mg/L (0 mg/L–10 mg/L). A diagnosis of methotrexate toxicity was established. Calcium folinate was immediately administered for rescue therapy, along with two sessions of hemoperfusion, recombinant human granulocyte colony-stimulating factor injection and recombinant human thrombopoietin injection to support hematopoiesis, and moxifloxacin for antimicrobial coverage. On 22 January 2025, his oral ulcers worsened, with bleeding, rupture, and hemorrhage from the nasal cavity and perianal region. He was unable to eat, and skin examination revealed dark erythematous patches and bullae. The repeat laboratory test demonstrated leukocytes of 0.09 × 10^9^/L, RBCs of 3.19 × 10^12^/L, and a platelet count of 23 × 10^9^/L, and the patient was then transferred to our hospital for further management.

**TABLE 2:** Laboratory test indicators.

Laboratory test indicator	Normal value	Local hospital		Our department											
		1.21	1.22	1.22	1.23	1.24	1.25	1.26	1.29	1.30	2.1	2.13	2.18	2.22	3.11
WBC (×109/L)	3.5–9.5	0.24	0.09	0.06	0.1	0.15	0.11	0.13	0.22	0.62	6.34	29.6	9.64	6.17	4.02
NEUT (×109/L)	1.8–6.3	0.05	0.04	0.04	0.03	0.01	0.04	0.02	0.02	0.39	5.5	26.06	7.61	4.27	2.34
LYMPH (×109/L)	1.1–3.2	0.15	0.04	0.02	0.05	0.07	0.05	0.09	0.16	0.19	0.64	0.89	0.37	0.32	0.98
MONO (×109/L)	0.1–0.6	0.00	0.00	0.00	0.01	0.01	0.02	0.02	0.02	0.03	0.11	2.45	1.44	1.33	0.38
EO (×109/L)	0.02–0.52	0.04	0.01	0.00	0.01	0.06	0	0	0.02	0	0.01	0	0.01	0.03	0.28
RBC (×1012/L)	4.3–5.8	3.2	3.19	3.47	3.08	3.06	2.76	2.74	2.6	2.46	2.85	2.21	2.14	1.93	2.24
HGB (g/L)	130–175	94	94	103	91	94	82	81	77	73	85	66	66	60	68
PLT (×109/L)	125–350	42	23	21	8	15	10	8	6	21	35	168	195	209	182
hs-CRP (mg/l)	0–10	231.09	N	>200.0	>200.0	>200.0	>200.0	>200.0	>200.0	>200.0	116.6	43.6	74.7	70.6	38.6
PCT (ng/ml)	0–0.1	N	N	N	32.17	N	28.15	N	10.72	N	6.41	N	5.87	6.06	2.05

WBC, white blood cell; NEUT, neutrophil; Lymph, lymphocyte; Mono, monocyte; EO, eosinophil; RBC, red blood cell; HGB, hemoglobin; PLT, platelet; HS-CRP, high-sensitivity C-reactive protein; PCT, procalcitonin; N, not tested.

**FIGURE 1:**
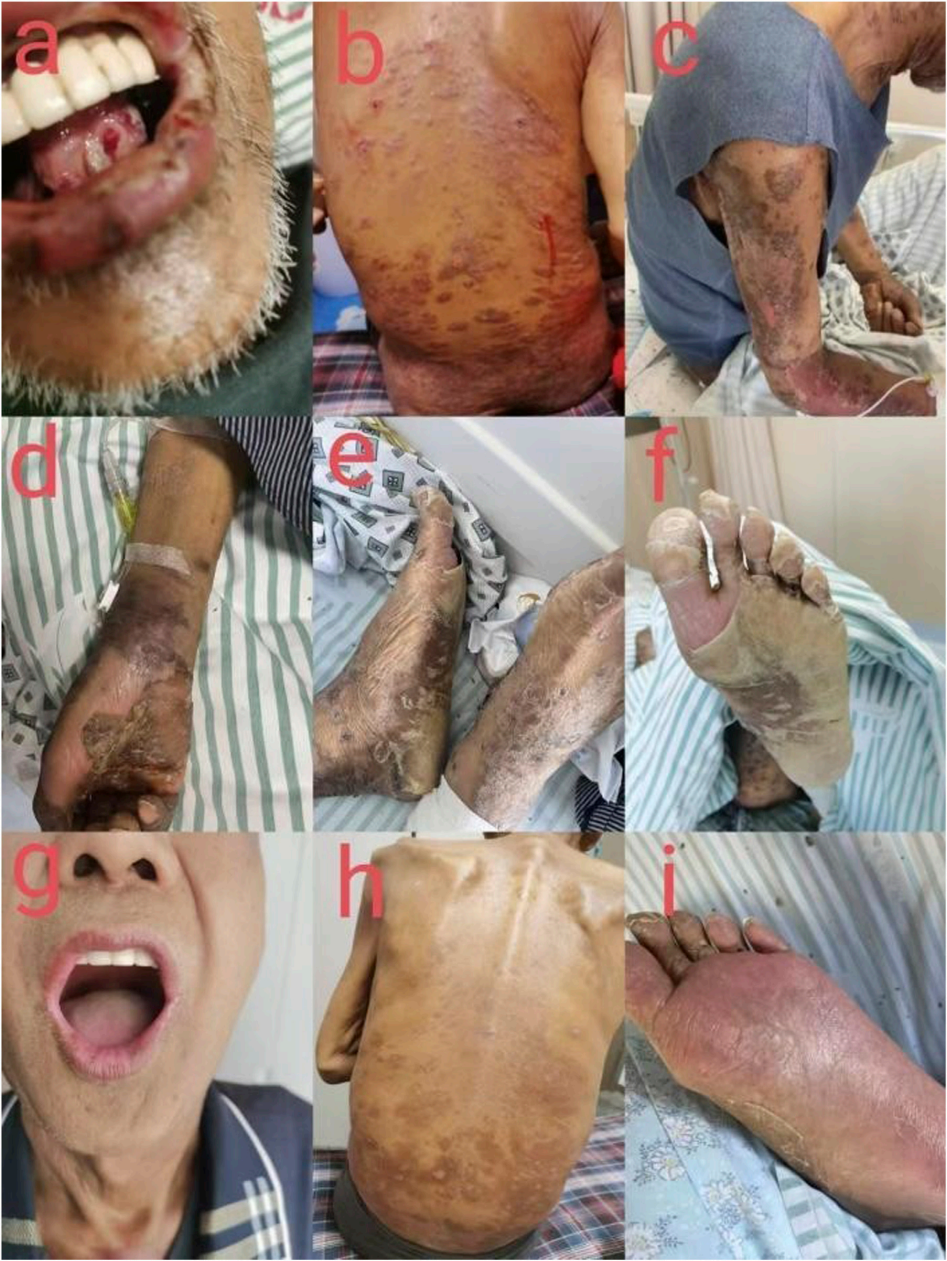
**(a)** Ulcers and erosion on the lips scattered at the point of bleeding; buccal mucosa and tongue were covered with white membranes. **(b)** Dark erythematous patches and bullae skin at the time of admission. **(c)** When admitted to the hospital, the skin showed dark erythematous patches and bullae and was partially ulcerated. **(d)** Day 28: peeling of palm epidermis. **(e)** Day 28: peeling of both feet. **(f)** Day 28: Peeling of the foot. **(g)** On the 51st day, the lips returned to a rosy hue and was without bleeding or ulcers. **(h)** On the 51st day, the dark erythema and bulla disappeared, scattered in pigmentation. **(i)** On the 51st day, necrotic skin was detached, epithelialized, and new skin was attached.

Upon hospital admission, the patient was conscious and exhibited febrile response, dysphagia, cough, wheezing, and respiratory distress. Physical examination revealed oral mucosal erosions (Figure 1), accompanied by facial edema, darkened skin, erythematous patches and bullae (Figure 1), fissuring on the palms (Figure 1), and perianal erosions. Vital signs included a body temperature of 38.9 °C; heart rate, 120 beats per minute; respiratory rate, 20 breaths per minute; and blood pressure, 186/98 mmHg. Auscultation revealed no abnormal findings in pulmonary or cardiac sounds, whereas Nikolsky’s sign was positive. Laboratory tests demonstrated leukopenia with a leukocyte count of 0.06 × 10^9^/L (3.5–9.5 × 10^9^/L), anemia with an erythrocyte count of 3.47 × 10^12^/L (4.3–5.8 × 10^12^/L), and thrombocytopenia with a platelet count of 21 × 10^9^/L (125–350 × 10^9^/L). Inflammatory markers were markedly elevated: high-sensitive C-reactive protein >200.0 mg/L (0 mg/L–10 mg/L), procalcitonin of 32.17 ng/mL (0 ng/mL–0.1 ng/mL), and fungal D-glucan detection (Table 3) >600.00 pg/mL (0 pg/mL–95 pg/mL). Microbiological analysis identified *Staphylococcus aureus* in sputum culture, with nucleic acid detection confirming methicillin-resistant *Staphylococcus aureus*. The thoracic computed tomography (CT) revealed scattered bilateral pulmonary infiltrates and multiple nodular opacities in both the lungs.

**TABLE 3:** Fungal D-glucan detection.

	Normal value	Day 2	Day 10	Day 15	Day 23
Fungal D-glucan detection (pg/m)	0–95	>600	207.57	90.88	58.734

Upon admission, we immediately monitored serum methotrexate concentrations, which returned at 0.225 μmol/L. The patient was diagnosed with methotrexate poisoning. Immediate intervention included calcium leucovorin (20 mg iv slow q6h) as an antidote, along with normal saline, sodium bicarbonate hydration, and urine alkalization to promote renal excretion of methotrexate. Hemoperfusion and continuous renal replacement therapy (CRRT) were used to enhance drug clearance. Supportive care included transfusions of RBCs and platelets, supplemented with recombinant human granulocyte colony-stimulating factor injection and recombinant human thrombopoietin injection to support hematopoiesis. Empirical broad-spectrum antimicrobial therapy was initiated with meropenem (lg iv. drip Q12H), vancomycin hydrochloride (50 mg iv. drip Q12H), and micafungin sodium (200 mg infusion pump QD). Adjunctive treatments included intravenous immunoglobulin and oral mucosal care with Kangfuxin liquid, a traditional Chinese medicine preparation extracted from *Periplaneta americana* (American cockroach), which is known to promote wound healing, reduce inflammation, and support tissue regeneration. On day 3, the skin bullae ulcerated and began to form scabs, and serum methotrexate levels decreased to 0.064 μmol/L. On day 5, epidermal desquamation was observed, accompanied by mucosal hemorrhagic spots and ulcerations.

On day 22, the patient’s erythema and bullae resolved, and extensive epidermal desquamation was present across the body. The necrotic skin tissue was shed progressively, and re-epithelialization commenced, leading to the regeneration of new epidermis.

**FIGURE 2:**
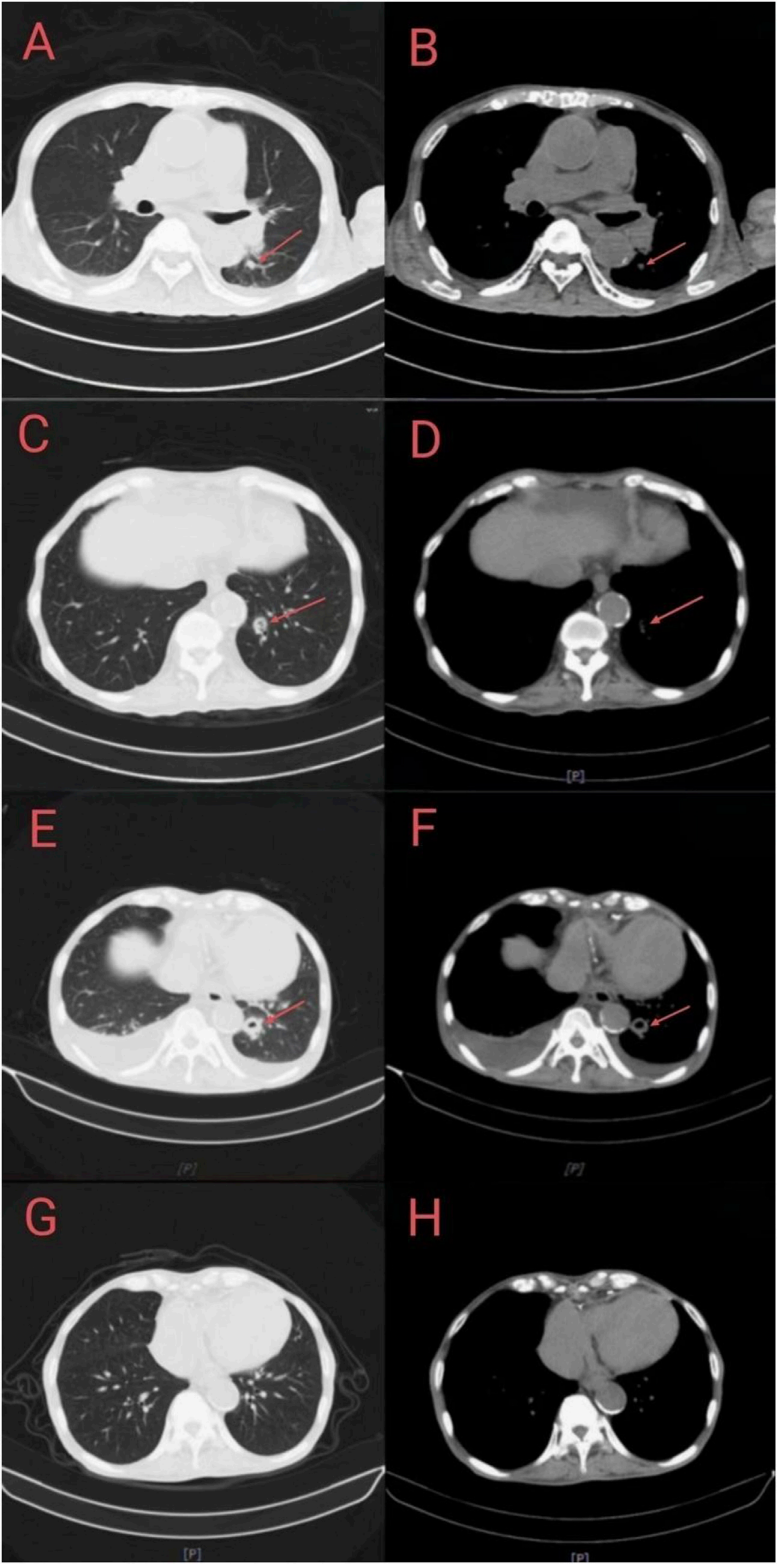
CT image of a 69-year-old male’s lung. **(A,B)** Chest CT scans at the time of admission showed that both lungs were scattered with inflammatory lesions, nodules were found in both lungs, and inflammatory nodules were seen on the lower left lobe. **(C,D)** Day 28: chest CT scan showed enlarged nodules in the lower lobe. **(E,F)** Chest CT scan on day 42 showed that the inflammatory nodule lesions in the lower lobe on the left lower lobe were slightly larger than those on the front, and there was a cavitary lesion. **(G,H)** Chest CT scan on day 54 of discharge showed a decrease in both lung nodules.

**FIGURE 3:**
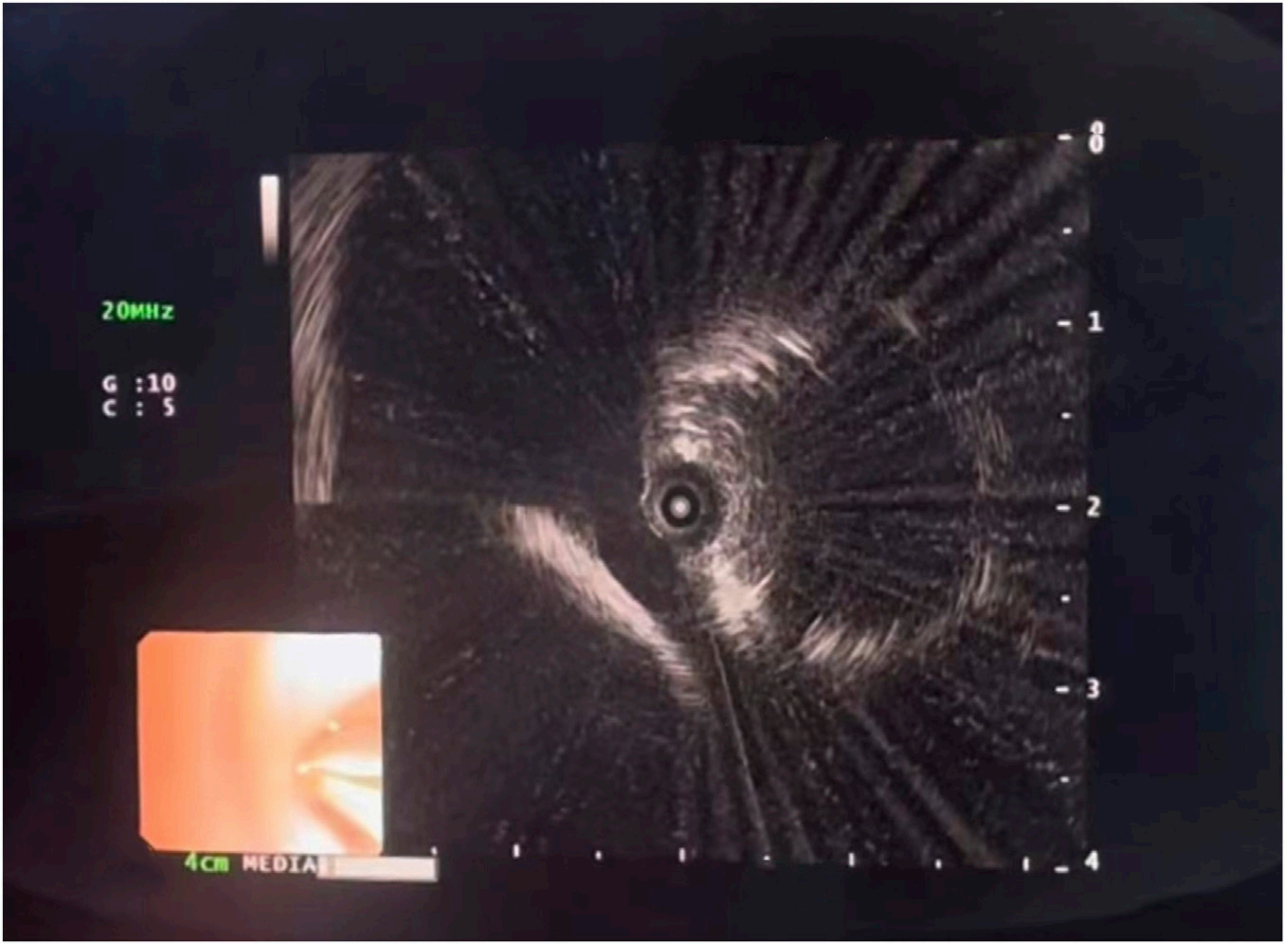
Blood vessel is encased or infiltrated by surrounding abnormal, heterogeneously hypoechoic tissue.

On day 28, the majority of the patient’s necrotic skin had desquamated, along with underlying skin regeneration. The thoracic CT scan (Figure 2) demonstrated an enlarging inflammatory nodule in the left lower lobe. The antifungal therapy was subsequently modified to voriconazole (0.2 g iv. drip Q12H). On day 36, persistent cough and dyspnea prompted a change in the antifungal regimen to isavuconazole (0.2 g PO QD). On day 42, follow-up thoracic CT demonstrated cavitation within the left lower lobe (Figure 2). Pathogen metagenomic next-generation sequencing (mNGS) of a respiratory specimen identified *Rhizopus microsporus*. Endobronchial ultrasound (EBUS) imaging showed vasculature encased by hypoechoic tissue, which is consistent with invasive pulmonary mucormycosis (Figure 3). The patient continued isavuconazole therapy.

On day 51, the patient’s lip coloration had returned to a rosy hue without bleeding, and the lip skin had scabbed and scattered pigmentations (Figure 1). Repeat laboratory tests showed a leukocyte count of 4.02 × 10^9^/L, an erythrocyte count of 2.24 × 10^12^/L, a platelet count of 182 × 10^9^/L, a high-sensitive C-reactive protein level of 38.6 mg/L, and procalcitonin of 2.05 ng/mL. Hematologic parameters were restored, and infection indices were reduced. So the patient was discharged on maintenance oral isavuconazole, with ongoing monitoring of hepatic and renal function, complete blood count, and other relevant parameters. The antifungal therapy was planned to be discontinued after a duration of 3–6 months.

At the 14-day follow-up after discharge, laboratory parameters included a leukocyte count of 3.73 × 10^9^/L, an absolute neutrophil count of 2.75 × 10^9^/L, an erythrocyte count of 2.24 × 10^12^/L, a platelet count of 84 × 10^9^/L, a high-sensitive C-reactive protein level of 63.68 mg/L, and a procalcitonin level of 0.42 ng/mL. By the 54-day follow-up, a repeat chest computed tomography demonstrated significant radiological improvement (Figure 2). The patient reported a marked clinical recovery. Follow-up after discharge indicated the absence of sequelae.

This study was approved by the appropriate Ethics Committee of Shandong University of Traditional Chinese Medicine Affiliated Hospital. Written informed consent was obtained from the patient’s family.

## 3 Discussion

In this case, the causative agent of toxicity is methotrexate. The patient with psoriasis receiving low-dose methotrexate for the first time developed adverse effects such as fever, mucocutaneous ulceration, and myelosuppression. Due to individual susceptibility, this patient had severe methotrexate toxicity, including mucocutaneous ulceration, extensive epidermal desquamation, myelosuppression, and systemic immunosuppression. The subsequent development of invasive pulmonary mucormycosis further complicated management, leading to a protracted disease course and complex clinical presentation. It carried a high mortality risk.

This clinical manifestation has similar features to severe drug eruptions and staphylococcal scalded skin syndrome. We require differential diagnostic evaluations. Severe drug eruptions are life-threatening mucocutaneous adverse reactions caused by pharmacologic agents. They represent the most severe end of the drug hypersensitivity spectrum. Typical types of severe drug eruptions include Stevens–Johnson syndrome (SJS), toxic epidermal necrolysis (TEN), and drug reaction with eosinophilia and systemic symptoms (DRESS). These conditions are characterized by mucosal erosions, erythematous and blistering skin lesions, fever, lymphadenopathy, and multi-organ involvement. Complications of severe drug eruptions often include secondary bacterial infections, sepsis, electrolyte imbalances, and organ failure. First-line management generally involves early high-dose systemic corticosteroids, intravenous immunoglobulin (IVIG), and supportive care, such as fluid resuscitation and anti-infective therapy. Staphylococcal scalded skin syndrome also shows extensive epidermal detachment, erythema, and blister formation. However, it is caused by exfoliative toxins produced by *Staphylococcus aureus*. This syndrome mainly affects pediatric populations, usually does not involve the mucous membranes, and often shows positive Nikolsky’s sign. Definitive diagnosis of staphylococcal scalded skin syndrome is made through bacterial cultures and histopathological analysis.

Studies have found that the main mechanism of methotrexate toxicity is the disruption of cellular metabolic processes (Bharadwaj et al., 2023). Methotrexate acts as a potent inhibitor of the enzyme dihydrofolate reductase. It prevents dihydrofolate from turning into tetrahydrofolate, and this process further reduces intracellular folate levels. Folate plays a critical role in nucleotide synthesis. For this reason, the inhibitory effect of methotrexate impairs the production of purines and pyrimidines, which are essential for cell division (Bedoui et al., 2019; Koźmiński et al., 2020). Cells with rapid division rates, such as those in the bone marrow and mucosal linings, are especially sensitive to this effect. This high sensitivity leads to adverse outcomes such as myelosuppression and mucositis (Yin et al., 2025). Pharmacokinetic studies report that methotrexate has an elimination half-life of 5 h–8 h. Its systemic clearance ranges from 4.8 to 7.8 L/h (Bannwarth et al., 1996).

Myelosuppression represents the predominant life-threatening adverse effect associated with low-dose methotrexate chemotherapy (Kivity et al., 2014). It disrupts hematopoietic processes, resulting in pancytopenia, which compromises immune function and increases vulnerability to infectious agents. These infections can further damage the bone marrow microenvironment by injuring hematopoietic stem cells, progenitor populations, or stromal components (Long et al., 2025), thereby perpetuating a deleterious feedback loop between myelosuppression and infectious complications (Keramati et al., 2025). Leucovorin is the specific antidote for methotrexate toxicity. As a folic acid analog, this substance is taken up by cells and transformed into tetrahydrofolate. It can effectively bypass the blockade of dihydrofolate reductase caused by methotrexate, thereby restoring the cellular capacity to synthesize DNA, RNA, and proteins (Chan et al., 2025). This restoration of tetrahydrofolate enables resumed synthesis of nucleic acids and proteins, facilitating cellular recovery (Song et al., 2021). The administration of leucovorin rescue (typically 15 mg/m^2^ every 6 h) should commence 24 h post-initiation of methotrexate infusion (Chan et al., 2025).

Although leucovorin can ease methotrexate toxicity, the drug’s accumulation inside the body may still lead to severe myelosuppression. Myelosuppression occurs in less than 1% of patients receiving low-dose methotrexate and is usually reversible when the drug is stopped, and it rarely puts life at risk (Kevin and Zipursky, 2019). For patients receiving methotrexate therapy, preventive folic acid supplementation is suggested. This is to lower the rate of these adverse effects. Studies show that a weekly folic acid dose of 5 mg–10 mg is an effective preventive approach. This dose is generally taken 24 h after methotrexate is given.

This dosing strategy stands as the optimal method for balancing therapeutic efficacy and safety. Exceeding this range may fail to deliver additional therapeutic benefits (Fraenkel et al., 2021). Thus, combining early clinical monitoring with preventive folic acid supplementation is key. It helps minimize the risk of methotrexate-induced toxicity.

This case describes a patient with severe psoriasis who received methotrexate treatment. The patient had impaired renal function, and methotrexate is mainly eliminated through the kidneys. Because renal function significantly affects pharmacokinetics, the patient was administered acid supplementation. Even so, the rapid appearance of severe toxicity indicated pharmacogenomic factors. Genetic polymorphisms probably worsened methotrexate accumulation, leading to life-threatening drug toxicity. Early clinical measures including leucovorin rescue therapy and hemoperfusion for detoxification were consistent with established management guidelines. The mucocutaneous lesion recovered gradually. Despite these interventions, the phase of severe immunosuppression and mucosal barrier damage led to opportunistic infections. These infections then developed into invasive pulmonary mucormycosis. The patient received timely diagnostic tests, such as CT, mNGS, and EBUS. These examinations facilitated targeted therapy, leading to the successful treatment of the infection. The patient was discharged from the hospital and regained the ability to handle daily activities independently.

Patient responses and toxicity profiles vary even with the same methotrexate dose. This variation is partly due to genetic polymorphisms. These polymorphisms may be related to the drug’s pharmacokinetics and pharmacodynamics, including processes such as absorption, metabolism, excretion, cellular transport, and interactions with effector targets (Schmiegelow, 2009). Idiosyncratic reactions can exacerbate the toxicity of bone marrow cells. The severity of myelosuppression here depends heavily on individual differences. Research has shown that polymorphisms in the methylenetetrahydrofolate reductase (MTHFR) gene, specifically the A1298C and C677T variants, play a role in the etiology of methotrexate-related toxicity. Both variants are associated with reduced MTHFR enzyme activity, which may elevate the risk of adverse effects (Ashton et al., 2009). Additionally, studies indicate that polymorphisms in the *SLCO1B1* gene are linked to the clearance and toxicity of methotrexate (Martinez et al., 2018). In summary, interindividual differences in response to methotrexate are substantial and are likely influenced by these genetic polymorphisms. Therefore, the application of pharmacogenetics and therapeutic drug monitoring may help optimize methotrexate therapy for improved safety and efficacy (Dervieux et al., 2005).

Traditionally, serum methotrexate concentration has been the most widely used indicator for therapeutic drug monitoring. Yet this method has built-in limitations when applied to low-dose treatment regimens. Methotrexate has a relatively short plasma half-life, so its concentrations vary greatly between doses. They often fall to levels that cannot be detected (van de Meeberg et al., 2023). As a result, serum concentrations cannot accurately show long-term drug accumulation. Methotrexate enters cells through folate transporters. Once it enters erythrocytes, it is extensively broken down into long-acting methotrexate polyglutamates (Belkov et al., 1999). Erythrocytes have a lifespan of approximately 120 days and no nuclei, so they cannot actively remove these metabolites. Thus, the concentration of methotrexate polyglutamates in RBCs (RBC-MTX-PG) is a more reliable measure of long-term drug exposure. It also better reflects tissue-level pharmacokinetics.

However, our hospital could not measure RBC-MTX-PG. Our assessment depended only on serum methotrexate concentrations, which failed to fully show the actual drug exposure. Measuring intracellular methotrexate polyglutamates in erythrocytes is a better method. It helps explain the variability in treatment response, predict and manage toxicity, and provides a scientific basis for dose adjustment. This approach is essential for achieving truly personalized therapy.

This case report describes a patient who experienced severe and rapidly progressive toxicity following the initial low-dose methotrexate administration. The patient later developed complications that were difficult to manage. This situation emphasizes the vital need to identify patients at risk of idiosyncratic reactions, as these reactions can trigger life-threatening methotrexate toxicity (Pitakkitnukun et al., 2024). Thus, this case highlights how important it is to assess the risk factors for severe drug-related adverse events.

In conclusion, we report a rare case of severe intoxication after using low-dose methotrexate. This case acts as an important reminder of the variability in populations with idiosyncratic reaction. Strengthening genetic testing, improving risk assessment for drug toxicity, and creating targeted risk prediction models are key steps to reduce risks. These measures boost the safety and efficacy of clinical pharmacotherapy. They form the foundation for personalized medicine and serve as an essential part of precision medicine.

## Data Availability

The original contributions presented in the study are included in the article/Supplementary Material; further inquiries can be directed to the corresponding authors.
